# Cyclophilin inhibition as a strategy for the treatment of human disease

**DOI:** 10.3389/fphar.2024.1417945

**Published:** 2024-07-08

**Authors:** Winston T. Stauffer, Asha Z. Goodman, Philippe A. Gallay

**Affiliations:** Department of Immunology & Microbiology, The Scripps Research Institute, La Jolla, CA, United States

**Keywords:** cyclophilin, cyclophilin inhibitors, human disease, human diseases, viruses, treatments

## Abstract

Cyclophilins (Cyps), characterized as peptidyl-prolyl *cis-trans* isomerases (PPIases), are highly conserved and ubiquitous, playing a crucial role in protein folding and cellular signaling. This review summarizes the biochemical pathways mediated by Cyps, including their involvement in pathological states such as viral replication, inflammation, and cancer progression, to underscore the therapeutic potential of Cyp inhibition. The exploration of Cyp inhibitors (CypI) in this review, particularly non-immunosuppressive cyclosporine A (CsA) derivatives, highlights their significance as therapeutic agents. The structural and functional nuances of CsA derivatives are examined, including their efficacy, mechanism of action, and the balance between therapeutic benefits and off-target effects. The landscape of CypI is evaluated to emphasize the clinical need for targeted approaches to exploit the complex biology of Cyps and to propose future directions for research that may enhance the utility of non-immunosuppressive CsA derivatives in treating diseases where Cyps play a key pathological role.

## 1 Introduction

Currently for a wide range of human diseases, medical treatment is limited to palliative care, preventative strategies and life-style alterations, or drug treatments of limited effectiveness. Patients suffering from these maladies, including but not limited to metabolic diseases like metabolic dysfunction-associated steatotic liver disease (MASLD) and its progression to metabolic dysfunction-associated steatohepatitis (MASH) (Petagine et al., 2023), and viral infections such as hepatitis C virus (HCV) (Manns and Maasoumy, 2022), human immunodeficiency virus (HIV) (Landovitz et al., 2023), or severe acute respiratory syndrome coronavirus 2 (SARS-CoV-2) (Lamers and Haagmans, 2022) are continually in need for new Food and Drug Administration (FDA)-approved drug treatments which can directly address or even reverse the causative factors, either as monotherapies or in combination with previous standard-of-care drugs. Though the molecular pathways governing these disorders can vary greatly, there are some highly conserved gene families that, due to their ubiquity and involvement in numerous cell functions, play an essential role in the initiation and progression of numerous otherwise unrelated diseases. Such a gene family are the Cyps, PPIases which, at their core, function to catalyze the transformation of peptide bonds at proline residues from *trans* isomers to the less common *cis* form. (Wang and Heitman, 2005; Kim et al., 2008; Nigro et al., 2013a; Elrod and Molkentin, 2013). This simple function is sufficient for Cyp family members to play roles nearly everywhere in and out of the cell, from the nucleus to the extracellular environment. Cyps function as protein chaperones, secondary messengers, regulators of membrane permeability, and more. Together, Cyps and their PPIase relatives are represented in most forms of life, including eukaryotes, bacteria, and archaea. It is thus unsurprising that Cyps are of interest as possible druggable targets in human disease, even including infectious diseases where host factors are involved. Indeed, a CypI drug was discovered before Cyps were. They are the mechanism behind the immune inhibitor CsA, a designated WHO Essential Medicine, for which the family is named. (Tedesco and Haragsim, 2012). Since then, numerous related CypI have been identified, which have been or are currently under investigation for the treatment of human disease. This review will serve as a comprehensive overview of the Cyp family, the diseases in which they play a role, and the drugs and strategies which aim to inhibit those roles.

## 2 Overview of PPIases and the cyclophilin family

PPIases embody a superfamily of chaperone enzymes, crucial for the dynamic process of protein folding, activation, and degradation within cellular environments. PPIase enzymes accomplish this by facilitating the cis-trans isomerization of proline peptide (Xaa-Pro) bonds in oligopeptides. (Dunyak and Gestwicki, 2016). Among the PPIases are the Cyp family, a highly conserved group of proteins found in virtually all organisms, including mammals, plants, insects, fungi, and bacteria. Variations in the N- and C-terminal regions of different Cyp family members, situated next to the common 109 amino acid or Cyp-like domain (CLD), dictate their specific subcellular localizations and biological functions. (Marks, 1996; Wang and Heitman, 2005). The observed diversity and ubiquitous nature of these chaperones underscore the versatility of Cyps across various biological systems. The three most characterized Cyp family members are summarized below and in Table 1.

**TABLE 1:** Cyclophilin localization, functions, and relation to human diseases.

Cyclophilins	Localization	Associated Diseases	Unique Functions
CypA	Cytosol	HIV-1 (Franke et al., 1994; Thali et al., 1994)	Immunomodulation, protein-folding, interacts with HIV-1 capsid, uncoating and nuclear import of viral genome, etc. (Towers et al., 2003)
		HCV (Nakagawa et al., 2005; Yang et al., 2008a; Kaul et al., 2009; Foster et al., 2011)	HCV RNA amplification and binding (Foster et al., 2011)
		Neurodegenerative e.g., Parkinson’s and Alzheimer’s	Implicated in a-synuclein aggregation (Favretto et al., 2020)
		Inflammation e.g., Rheumatoid Arthritis (Billich et al., 1997)	MMP Activation (Yang et al., 2008b)
CypB	Endoplasmic reticulum	MASH Osteogenesis Imperfecta and HERDA (equine) (Rios et al., 2005)	Folding and export of collagen Collagen folding, prolyl 3-hydroxylation, and lysine hydroxylation of collagen Collagen Processing (Terajima et al., 2019)
CypD	Mitochondria	Myocardial infarction	Cell death mediation via mPTP opening (Lim et al., 2011)
		Neurodegenerative e.g., Alzheimer’s	Cellular and synaptic perturbations via mPTP opening (Cohen and Taraboulos, 2003)

### 2.1 Cyclophilin A (CypA)

**FIGURE 1:**
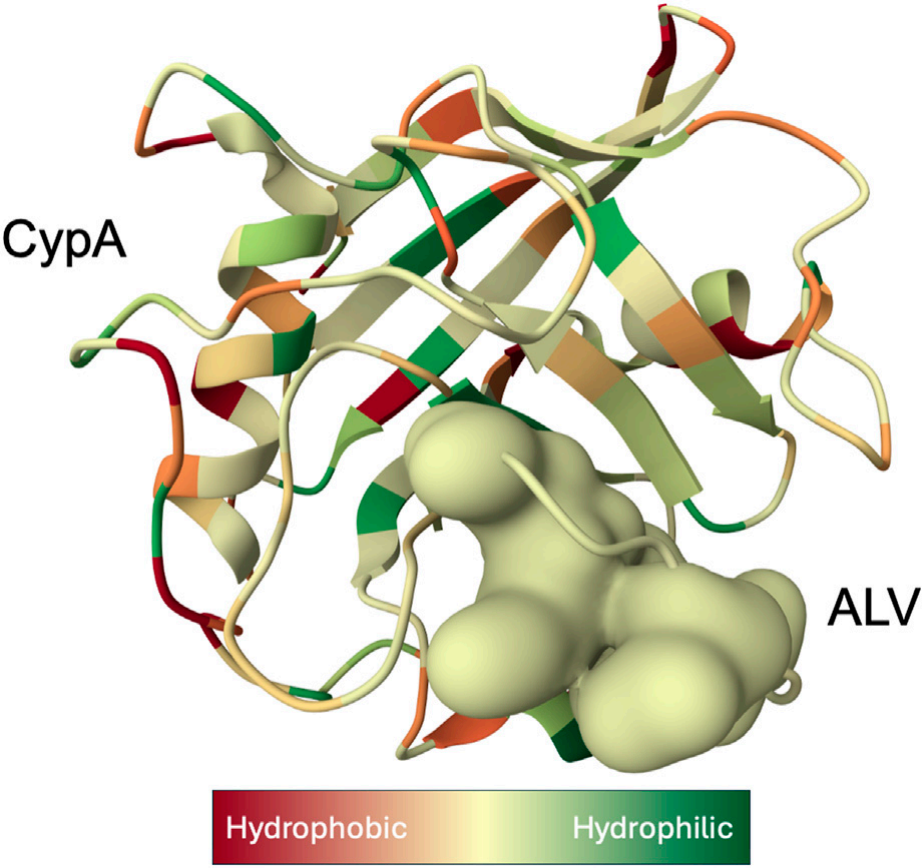
X-ray structure of the Cyclophilin A (CypA)-Alisporivir (ALV) complex determined at 1.5 Å resolution (Dujardin et al., 2018). CypA structure is displayed as a ribbon with secondary-structure elements visualized. Bound ALV is depicted as a coarse Gaussian surface. The hue of the molecular complex transitions from shades of red to green to represent hydrophilic and hydrophobic properties, respectively, and was prepared with Molstar Viewer (Sehnal et al., 2021).

As a housekeeping protein and the first of the 18 cyclophilins identified to date, the PPIase A, also known as CypA, accounts for approximately 0.4% of the complete intracellular proteome. (Wang and Heitman, 2005). Human CypA is characterized by an eight-stranded anti-parallel β-barrel structure, with two α-helices on both sides, with the loop from Lys118 to His126 and 4 B strands (B3-B6) (Figure 1). (Kallen et al., 1991). The hydrophobic core, responsible for its PPIase activity, serves as the binding site for both CsA (Fesik et al., 1990), and the HIV-1 Gag polyprotein (Braaten et al., 1997).

#### 2.1.1 CypA: Cytosolic roles

Intracellular CypA plays an important role in numerous human diseases including neurodegeneration. For example, CypA performs a protective function against protein misfolding diseases, highlighted by the significant enrichment of CypA in the insoluble fraction of spinal cord tissues from patients with amyotrophic lateral sclerosis. (Pasetto et al., 2021). Highly expressed in the central nervous system, work by the Zeckstetter lab shed light on how CypA’s ability to bind to two sites of α-synuclein (α-syn), an intrinsically disordered protein, is disrupted by a mutation of alanine 53 to glutamate. The resulting inability of CypA to assist in α-syn conformational changes may lead to the pathological aggregation of the protein, as observed in patients with early-onset Parkinson’s disease. (Favretto et al., 2020). Indeed, knocking out CypA in an alternative mouse study resulted in neurodegenerative disease presenting as frontotemporal dementia. (Pasetto et al., 2021).

**FIGURE 2:**
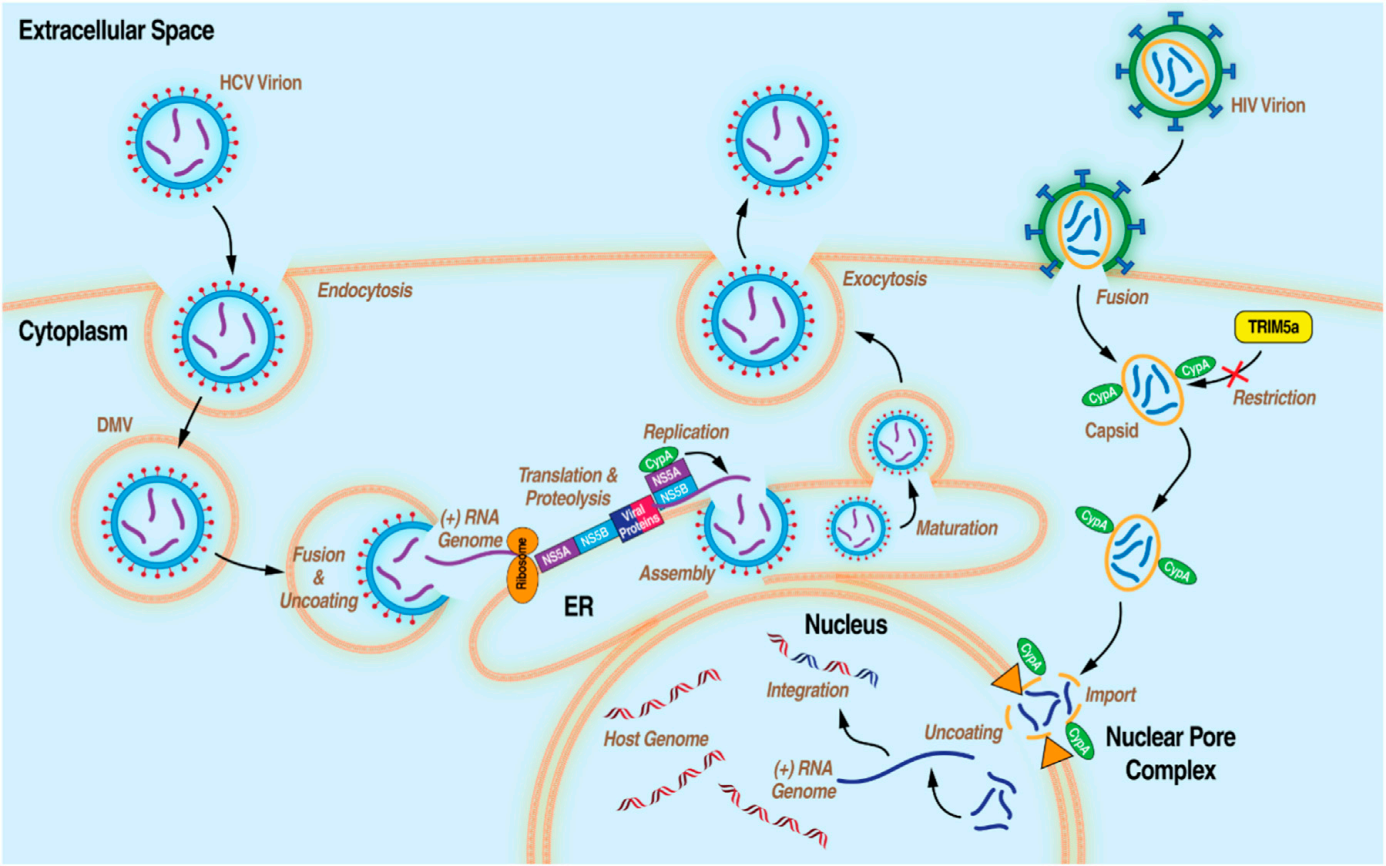
Diagram of the life cycles of HCV and HIV-1 with the processes that CypA has been demonstrated to be essential.

In addition to its roles in humans, cytosolic CypA plays key roles in the replication of prime human pathogens such as HIV-1 and HCV (Figure 2). In the current model for the role of CypA in HIV-1 infection, cytosolic CypA present in infected cells binds directly to a proline-rich domain of the HIV-1 capsid that forms the shell (or core) that surrounds and protects the viral genome during its nuclear transport. (Kane et al., 2018). CypA facilitates the docking of the viral core onto the nuclear membrane, the uncoating of the viral genome, and ultimately to the passage of the viral genome through the nucleopore allowing the integration of the HIV-1 genome into the host chromosomes (De Iaco and Luban, 2014; Selyutina et al., 2020). Another milestone study treated a human T cell line (Jurkat) and primary human CD4^+^ T cells with CsA to demonstrate CypA provides protection of the HIV-1 core by inhibiting the antiretroviral activity of the human tripartite motif (Selyutina et al., 2020). In the present conceptualization of CypA’s involvement in HCV infection, cytosolic CypA binds directly to the HCV nonstructural protein 5A (NS5A) to mediate its *cis-trans* isomerase activity in a prolyl-rich region of the domain II of NS5A (Coelmont et al., 2010; Rosnoblet et al., 2012). The interaction between CypA and NS5A is critical for the formation of double membrane vesicles (DMVs) derived from the ER where HCV amplifies its viral genome in a compartment protected from viral RNA immune host sensors. (Chatterji et al., 2015; Romero-Brey et al., 2015; Wolff et al., 2020).

#### 2.1.2 CypA: Roles in the immune response

Secretory CypA is a pro-inflammatory factor that attracts innate immunity cells (granulocytes, macrophages, and dendritic cells) to the inflammation site and mediates the pathogenesis of various diseases (Nigro et al., 2013b). CypA acts as a chemoattractant for stem cells, immature granulocytes, and the progenitors of dendritic cells, macrophages, T- and B-lymphocytes. (Khromykh et al., 2007). In this regard, CypA takes part in regenerative processes but most importantly in the immune response. CypA regulates the action of other chemokines and the production of pro-inflammatory cytokines (Dawar et al., 2017). CypA was shown to induce the differentiation and maturation of dendritic cells, and to enhance antigen uptake and presentation by these cells (Bharadwaj et al., 2004). Therefore, CypA can modulate both the innate and the adaptive immunity.

Secreted CypA as proinflammatory agent acts through its interaction with the cell surface receptor CD147 that initiates signaling cascades leading to extracellular signal-regulated kinase activation. This process is crucial for the chemotactic activity of CypA and involves specific residues in both CypA and CD147 (Yurchenko et al., 2002). The presence of elevated extracellular CypA levels is noted in various conditions, including rheumatoid arthritis, endometrial carcinoma, coronary artery disease, vascular smooth muscle (VCMC) diseases, diabetic kidney disease, and severe sepsis, marking it as a potential biomarker. Secretion of CypA, primarily by VSMCs and macrophages, is triggered in response to specific stimuli including reactive oxygen species (ROS) and lipopolysaccharides (LPS) and serves as a mediator of vascular tissue damage. (Sherry et al., 1992; Jin et al., 2004). Particularly, macrophages release CypA upon LPS stimulation or high glucose exposure, linking ROS to the pathogenesis of conditions such as atherosclerosis, hypertension, and restenosis. It should be noted these diseases are characterized, in part, by the promotion of VSMC proliferation.

CypA’s interaction with CD147 on leukocytes, which is upregulated in inflamed tissues, is crucial for the recruitment of T lymphocytes to these areas. This process is enhanced by the ability of activated T cells to migrate more efficiently, even in the absence of heparan sulfate receptors. (Damsker et al., 2007). Additionally, CypA facilitates extracellular matrix (ECM) degradation by inducing degranulation and stimulating matrix metalloproteinases (MMPs) expression in endothelial cells, macrophages, and monocytes. The inducement of degranulation is vital for ECM remodeling and the maintenance of vascular integrity. Notably, CypA, upon release from activated platelets, engages with various cell types including endothelial and immune cells, further influencing vascular and immune responses. It also plays a regulatory role in calcium homeostasis within the sarcoplasmic/endoplasmic reticulum through its interaction with the sarco(endo)plasmic reticulum Ca(2+) ATPase 2b (SERCA2b). (Seizer et al., 2014; Rath et al., 2020).

#### 2.1.3 CypA modifications: Overexpression, knockouts (KO), and single nucleotide polymorphisms (SNPs)

Both *in vitro* and *in vivo* CypA modification studies have shed light on the intricate mechanisms through which CypA influences disease progression, particularly in the context of cardiovascular health and viral infections. In cardiovascular research, CypA knockout (Ppia −/−) models have demonstrated a crucial role in modulating angiotensin II-induced cardiac hypertrophy: Ppia −/− mouse show a marked reduction in cardiac hypertrophy, pointing towards CypA’s involvement in the direct potentiation of ROS production, which in turn stimulates cardiac fibroblast proliferation and cardiac myocyte hypertrophy. (Satoh et al., 2009). One *in vitro* study by the Luban group demonstrates how tripartite motif protein 5α (TRIM5α), an RNA virus restriction factor, is antagonized by CypA. The restriction activity of TRIM5α, a member of the tripartite motif protein family, was first identified in 2004 as a potent inhibitor of HIV-1 and other retroviruses. TRIM5α exerts its effects by recognizing and inactivating the capsid shell of invading retroviruses, including HIV-1. (Ganser-Pornillos and Pornillos, 2019). Through a series of experiments involving T-REx-293 cells engineered to lack CypA revealed CypA as a pro-viral factor. (Zhao et al., 2023). Subsequent research of certain SNPs encoded in CypA, particularly in the promoter region, are linked to faster HIV-1 disease progression. The SNPs in CypA not only affect CD4^+^ T-cell decline in African Americans but also show a correlation with accelerated AIDS progression in European Americans, indicating a significant genetic component to individual susceptibility to HIV-1. (An et al., 2007).

#### 2.1.4 CypA, calcineurin pathway and T-Cell activation

Since CypA was originally identified as the primary cytosolic binding protein of the immunosuppressive drug cyclosporin A (CsA) in purified from bovine thymocytes (Handschumacher et al., 1984). T-cell activation is an essential physiological process in the immune response, initiated when T-cell receptors (TCRs) recognize antigens presented by antigen-presenting cells. This recognition triggers a cascade of intracellular signals, leading to an increase in intracellular calcium levels, which is essential for activating transcription factors such as nuclear factor of activated T-cells (NFAT). NFAT’s activation and nuclear translocation are pivotal for the transcription of genes involved in T-cell proliferation, differentiation, and the secretion of cytokines like interleukin-2 (IL-2) and neuron signaling molecules. Historically, CsA has been extensively used as an immunosuppressant in patients undergoing organ transplantation, leveraging its ability to diminish T-cell response to prevent graft rejection. (Starzl et al., 1981). The mechanism behind CsA’s immunosuppressive effects involves a ternary complex consisting of CsA, CypA, and calcineurin (CaN). Although all members of the Cyp family exhibit PPIase activity, it is the binding and subsequent inhibition of CaN that diminishes the efficacy of the human immune response. (Liu et al., 1991). CaN is a ubiquitous serine/threonine phosphoprotein phosphatase dependent on Ca^2+^ and the calcium-sensing protein calmodulin (CaM). The increase in intracellular calcium levels upon TCR engagement ultimately leads to CaN activation and subsequent dephosphorylation of NFAT. CaN also dephosphorylates the myocyte-specific enhancer factor 2 (MEF2), forkhead (FOXO), and EB (TFEB) transcription factors, all being involved in autophagy. (Lapierre et al., 2015). The charges and hydrophobic regions of the CsA-CypA drug-protein complex aligns with the CaN binding site, blocking the phosphatase activity and subsequent signal transduction in activated T-cells.

### 2.2 Cyclophilin B (CypB)

CypB localized within the endoplasmic reticulum (ER), plays a pivotal role in the protein folding process integral to the secretory pathway. CypB is known to be constitutively secreted by certain tissue-resident cells, such as fibroblasts and chondrocytes, and is recorded to contribute to the extracellular matrix composition. CypB bound to the cell surface via heparan sulfate proteoglycans is released by matrix metalloproteinases (MMPs). (Rycyzyn et al., 2000). Additionally, CypB’s expression is notably upregulated in malignant glioma, suggesting its involvement in tumorigenesis and cancer progression. (Choi et al., 2014). The expression of CypB is also induced by ER stress, with overexpression shown to mitigate ER stress-induced cell death *in vitro*, indicating a protective function of CypB against cellular stress. (Kim et al., 2008).

#### 2.2.1 CypB: Unfolded protein response

CypB is integral to the ER stress response and the unfolded protein response (UPR), essential processes for maintaining proteostasis. (Liu et al., 1991). established that a deficiency in CypB precipitates ER stress and disrupts the normal UPR pathway. Within the context of neurodegenerative disorders, CypB’s importance is further emphasized by the work of Oh et al. (Oh et al., 2011), who demonstrated the overexpression of CypB mitigates neuronal apoptosis in Alzheimer’s disease models, particularly in scenarios characterized by the aggregation of amyloid proteins. This finding suggests a neuroprotective capacity of CypB, potentially offering a therapeutic strategy for Alzheimer’s disease through the modulation of CypB expression. Additionally, research conducted by Ram and Ramakrishna (Ram and Ramakrishna, 2014) on HeLa cells revealed that CypB KO results in ER stress and UPR activation, leading to a non-apoptotic form of cell death known as paraptosis, characterized by significant cytosolic vacuolation.

#### 2.2.2 CypB: Role in collagen secretion

CypB is integral to the biosynthesis of collagen, catalyzing the isomerization of peptide bonds within the procollagen triple helix, an activity regulated by polyphosphate (PolyP). (Ishikawa et al., 2012; Khong et al., 2020). The dysfunction or absence of CypB has been implicated in various pathological conditions, including Hereditary Equine Regional Dermal Asthenia (HERDA), a genetic skin disorder in horses characterized by hyperextensible skin and pronounced scarring (Tryon et al., 2005), and osteogenesis imperfecta (OI) in mice, a condition that arises when the Prolyl 3-Hydroxylase 1/Cartilage-associated Protein/Cyclophilin B (P3H1/CRTAP/CypB) complex is compromised (Choi et al., 2009). The P3H1/CRTAP/CypB complex is essential for the proper folding and maturation of collagen molecules; disruptions in this complex can lead to the accumulation of improperly folded procollagen, which may form insoluble, cytotoxic aggregates that are typically removed via autophagy. Mutations affecting CypB can interfere with this protective mechanism, resulting in the accumulation of defective collagen and contributing to the manifestation of related diseases. Furthermore, CypB plays a critical role in the retro translocation of misfolded proteins from the ER back to the cytosol for degradation.

#### 2.2.3 CypB: KO mouse characterization

The characterization of CypB KO mice has unveiled specific physiological roles and disease associations of CypB, differentiating its functions from CypA. Aside from moderate osteoporosis in older ages, the absence of CypB does not impact the overall viability of mice and is generally well-tolerated. (Choi et al., 2014). This indicates that while CypB contributes to bone health, it is not essential for survival. Moreover, the role of CypB in bone development disorders, notably osteogenesis imperfecta (OI), has been researched: the Choi group found that severe OI develops in CypB KO mice, indicating the importance of the P3H1/CRTAP/CypB complex in collagen formation. The absence of CypB disrupts collagen processing, particularly affecting procollagen’s localization to the Golgi apparatus and significantly reducing P3H1 levels. This disruption suggests that CypB’s involvement in procollagen retrotransport to the Golgi apparatus is critical for normal collagen formation and bone development. (Choi et al., 2009; Choi et al., 2014; Ram and Ramakrishna, 2014; Khong et al., 2020). Furthermore, the loss of CypB function, potentially induced by CsA, has been linked to the development of prion disease through the stabilization of long-lived aggresomes. (Cohen and Taraboulos, 2003). The Gallay lab recently demonstrated that mice lacking CypB, unlike those lacking CypA, were protected from non-alcoholic steatohepatitis (NASH) in both dietary and chemically induced models. (Stauffer et al., 2024). The resulting disruption of collagen folding and export, a central facilitation of liver fibrosis, is the predicted mechanism protecting mice from the development of NASH.

### 2.3 Cyclophilin D (CypD)

CypD encoded by the PPIF gene, is a mitochondrial-specific member of the Cyp family and is instrumental in controlling the mitochondrial permeability transition pore (mPTP). In scenarios of excessive calcium or phosphate and heightened intracellular oxidative stress, mitochondria release calcium via the mPTP in a process that does not involve transporters. The formation of the mPTP is ultimately triggered by calcium overloads, pro-apoptotic factors, and oxidative stress, and is further facilitated by CypD. CypD’s role in modulating the mPTP is highly relevant in the pathogenesis of a variety of conditions, including metabolic disorders such as metabolic dysfunction-associated steatotic liver disease (MASLD) and its progression to MASH (formerly non-alcoholic fatty liver disease (NAFLD) and NASH and eventual hepatocellular carcinoma (HCC). (Li et al., 2022). Diseases linked to CypD not related to metabolic disorders can be neurodegenerative. In Alzheimer’s disease, amyloid-beta, a hallmark of the condition, binds to CypD, leading to an increase in both the accumulation and production of mitochondrial ROS. This interaction not only recruits more CypD to the mitochondrial inner membrane but also heightens the mPTP’s susceptibility to calcium overload. Consequently, this increased vulnerability facilitates the opening of mPTP, resulting in the loss of mitochondrial membrane potential and ultimately cell death. (Du et al., 2008).

#### 2.3.1 CypD: Expression and KO

The metabolic function of CypD has been uncovered from several ablation studies; CypD has been implicated in the regulation of the mitochondrial acetylome, influencing outcomes post-myocardial infarction and contributing to heart failure. (Nguyen et al., 2013). However, results from several clinical trials (CIRCUS, NCT01502774; CYCLE, NCT01650662) suggested that the administration of CsA has no cardioprotective effect in humans, it neither reduced the burden of reperfusion injury nor improved clinical outcomes. (Cung et al., 2015; Ottani et al., 2016). Furthermore, altering the expression levels of CypD can modulate the sensitivity of cancer cells to apoptotic signals; increasing CypD expression enhances, whereas decreasing its expression reduces, tumor cells’ susceptibility to apoptosis. (Talari et al., 2018; Zhang et al., 2022). Despite these varied impacts on disease models and stress responses, CypD KO mice develop without apparent anomalies. It should be noted that positive outcomes associated with CypD knockouts are also recorded. For example, deficiency of CypD has been shown to mitigate mitochondrial disturbances, enhancing learning and memory capabilities in Alzheimer’s disease models. (Du et al., 2008). CypD’s absence confers resistance to oxidative stress-induced calcium perturbations in dendritic mitochondria (Sui et al., 2018) and been shown to protect mouse kidneys from CsA-induced damage, suggesting a protective buffer against CsA’s nephrotoxic effects (Palma et al., 2009; Klawitter et al., 2019). Furthermore, CypD KO mice exhibit resistance to ischemia/reperfusion-induced cardiac injury, with a marked reduction in cell death, contrasting with the detrimental effects observed when CypD is overexpressed, which leads to mitochondrial swelling and spontaneous cell death. (Sui et al., 2018). Therefore, the somatic localization of CypD should be critically considered when inhibiting the PPIase. Mice lacking collagen VI display a myopathic phenotype associated with ultrastructural alterations of mitochondria and sarcoplasmic reticulum, mitochondrial dysfunction with abnormal opening of the mPTP and increased apoptosis of muscle fibers. Importantly, the KO of CypD rescues the mitochondrial defects and prevents apoptosis in collagen VI-myopathic mice. (Palma et al., 2009).

## 3 Cyp Inhibition in Disease Treatment

### 3.1 Cyclophilin inhibitors (CypI)

As previously noted, Cyps were first discovered as the mechanism behind the immunosuppressant CsA. (Wang and Heitman, 2005; Nigro et al., 2013a). Like CsA, all subsequent drugs targeting Cyps have been inhibitor compounds (CypI) which appear to block the activity of all known Cyp family members with equal potency. CypI can be broadly categorized into two separate classes based on the original molecule they were based on. CsA derivatives are all variations on the 11-amino acid ring found in wild-type CsA produced by *T. inflatum*. (Borel et al., 1976). These include Alisporivir(ALV)/DEB025 (Paeshuyse et al., 2006; Coelmont et al., 2009; Zeuzem et al., 2015), Rencofilstat/CRV431/CPI-431–32 (Gallay et al., 2015; Trepanier et al., 2018; Kuo et al., 2019a), NIM811 (Traber RK et al., 1994; El Baradie et al., 2021), SCY635 (Hopkins et al., 2012a; Hopkins et al., 2012b), and others. Another class is based on sanglifehrin A, a peptide produced by the soil bacterium *Streptomyces* sp. *A92-308110*. (Zenke et al., 2001; Clarke et al., 2002). This compound, characterized in 1999, is also a natural immunosuppressant due to its inhibition of the interaction between CypA and CaN, much like the structurally distinct CsA. Sangliferhin A-derivatives, also called sangamides, include sanglifehrin B-D and NV556. (Gallay, 2012; Kuo et al., 2019b). While sangamides have been shown to bind all known Cyp family members, they have generally been shown to do so with lower affinity relative to CsA derivatives. Select individual representatives of both of these classes are outlined below and summarized in Table 2.

**TABLE 2:** Cyclophilin inhibitors in preclinical and clinical studies.

Cyp inhibitors	Class	Investigated therapeutic roles	Furthest clinical advancement
Cyclosporine A (Ciclosporin, CsA)	N/A	Autoimmune disease, transplant rejection, HCV and HIV infection, many others	Phase IV (Canadian Multicentre Transplant Study, 1983)
Sangliferhin A	N/A		N/A
Alisporivir (DEB025)	CsA-analog	HCV and HIV infection	Phase III (Zeuzem et al., 2015)
MM248	CsA-analog	HCV and HIV infection	N/A
NV556	Sangamide	HCV and HIV infection; MASLD/MASH; HCC	N/A
NIM811	CsA-analog	HCV and HIV infection	Phase IIa (Lawitz et al., 2011)
SCY635	CsA-analog	HCV and HIV infection	Phase IIa (Hopkins et al., 2012a)
Rencofilstat (CRV431)	CsA-analog	HCV and HIV infection; MASLD/MASH; HCC	Phase IIa (Harrison et al., 2022)

#### 3.1.1 ALV/DEB025

ALV (formerly known as DEB025) is a synthetic analog compound of CsA produced by Debiopharm and Novartis. The CsA 11-amino acid ring has been modified to have a methyl-alanine substitution at position 3 and an N-ethyl leucine substitution at position 4. These modifications enhance binding affinity to Cyps while abolishing the immunosuppressant effect by preventing the ALV-CypA complex from binding with calcineurin in T-lymphocytes. (Paeshuyse et al., 2006; Coelmont et al., 2009). ALV is well tolerated in patients, with the main side effect being a transient increase in serum bilirubin concentration at doses above 1000 mg. However there have been no other reports of elevated serum liver enzymes or other indicators of liver toxicity. (Zeuzem et al., 2015). Initially created as a potential treatment for HIV-1, ALV was found to be a more potent inhibitor of HCV replication and has since been primarily researched as an anti-HCV agent (see below). Advancing to Phase II trials, ALV was investigated both alone and along with FDA-approved anti-HCV agents PEG-interferon-α2a (PEG-IFN-α2a) and ribavirin. In a Phase IIa study with ALV and PEG-IFN-α2a, significant reductions in viral load were observed in patients with HCV genotypes 1, 4, and especially 2 and 3. Additional Phase II studies of a triple therapy of ALV together with PEG-IFN-α2a and ribavirin showed significant improvement over PEG-IFN-α2a and ribavirin alone. (Barve et al., 2015; Buti et al., 2015; Pawlotsky et al., 2015). Several cases of acute pancreatitis were observed however, including one death, leading to the cessation of the trial. (Zeuzem et al., 2015). It is unknown whether ALV alone or in combination with the other drugs contributed to the mortality. ALV has since been investigated as a drug treatment for hepatitis B virus (HBV) infection (Phillips et al., 2015), SARS-CoV2 infection (Pawlotsky, 2020; Softic et al., 2020; Dubinin et al., 2022), Duchenne’s muscular dystrophy (Schiavone et al., 2017; Dubinin et al., 2021), and Alzheimer’s disease (Montagne et al., 2021) (see below).

#### 3.1.2 MM284

Because CypA and other Cyps are known to be secreted to play significant extracellular roles, a new class of CypI was created to be cell-impermeable, thus isolating the effects of Cyp loss-of-function outside the cell. One example, MM284, is a CsA analog which has been altered with a glutamic acid–glycine–OH at position 6. This strongly negatively charged moiety prevents any affinity for the phospholipid membrane and thus prevents import into the cell while preserving the Cyp binding motif. (Heinzmann et al., 2015). MM284 has been used to uncover the role of CypA interaction with CD147 in conferring inflammatory signaling. Using these tools, extracellular CypA has been shown to be a viable therapeutic target in chronic inflammatory diseases, particularly those featuring leukocyte recruitment such as lung inflammation and myocarditis. (Seizer et al., 2015).

#### 3.1.3 NV556

Created by a combination of semisynthetic chemistry and bioengineering, NV556 is a non-immunosuppressive sangliferhin A analog. NV556 is potentially more useful than sangliferhin because it has fewer off target effects inhibiting drug transporters, as well as a higher barrier to the generation of drug resistance in viruses like HCV, hepatitis B virus (HBV), and HIV-1. (Hansson et al., 2015). NV556 has additionally been extensively characterized in preclinical studies as an anti MASLD/MASH agent, capable of reversing disease even after the development of liver inflammation and fibrosis. Remarkably, NV556 has also been shown to blunt the formation and prevalence of hepatocellular carcinoma (HCC) nodules downstream of severe MASH. (Kuo et al., 2019b; Simon Serrano et al., 2019). This, along with the CsA-analog rencofilstat (see below), represents the first demonstration of Cyp inhibition as a viable therapeutic avenue for the treatment of metabolic liver disease.

#### 3.1.4 NIM811

One of the first of its kind of non-immunosuppressive CsA analogs, NIM811 was isolated from fermented *T. inflatum*, the same fungi which produces CsA, and was found to have an N-methyl isoleucine substitution at the 4 position in the CsA ring. (Traber RK et al., 1994; Billich et al., 1995). Like the substitutions in similar drugs like ALV, this has the effect of disrupting the calcineurin-binding domain, while preserving or enhancing binding affinity to Cyps. NIM811 was originally isolated as part of an investigation into whether CsA or its derivatives would be potent anti-HIV agents due to their expected immunosuppressive effects. (Rosenwirth et al., 1994). While found to be non-immunosuppressive, NIM811 was nevertheless even more effective at preventing HIV replication. Subsequent studies also showed it had promise as an anti-HCV agent *in vitro*, similar to other CsA derivatives. (Ma et al., 2006). While NIM811 is a pan-Cyp inhibitor, it has mostly been characterized for its inhibition of CypD and its downstream effects on the opening of the mPTP. A Phase II trial was conducted treating patients with chronic HCV infection with NIM811. (Lawitz et al., 2011). While NIM811 alone did not produce a significant anti-viral effect, it was well tolerated in patients and NIM811 in combination with the PEG-interferon-α2a and ribavirin standard of care was more effective than the standard of care (SOC) alone. More recently, in addition to its antiviral properties, NIM811 has also shown promise as a treatment for *B. pertussis* infection (Ernst et al., 2018; Ernst et al., 2021), ischemia/reperfusion muscle injury (El Baradie et al., 2021), muscular dystrophy (Zulian et al., 2014), and Parkinson’s disease (Zhang et al., 2020).

#### 3.1.5 SCY635

Another derivative of CsA, SCY635 features a dimethylamino-ethylthio substitute at the alpha carbon on the sarcosine at the 3 postion and a hydroxyl substitute at the gamma carbon on the 4 N-methyl leucine. As with other non-immunosuppressive CsA derivatives, these modifications disrupt the CaN binding domain without impacting the Cyp binding domain. (Hopkins et al., 2012a; Hopkins et al., 2012b; Hopkins et al., 2012c). Phase I (Hopkins et al., 2012b)and IIa (Hopkins et al., 2012a) clinical trials in patients with chronic HCV showed decreased detectable HCV RNA and increased interferon activity, suggesting boosting the innate immune response was in part responsible for its antiviral activity.

#### 3.1.6 Rencofilstat

Developed by Hepion Pharmaceuticals, rencofilstat (formerly CRV431 or CPI-431–32) is a non-immunosuppressive CsA derivative that has been modified on amino acids 1 and 3 to abolish CaN interaction (and thus immunosuppression) and to enhance affinity for Cyps. Like CsA and other CsA derivatives, rencofilstat has poor solubility in water and must be suspended in a self-microemulsifying drug delivery system in order to be useful as a pharmaceutical. (Trepanier et al., 2018). Evidence shows it is effective against HBV infections (Trepanier et al., 2017; Gallay et al., 2019) and is slightly more effective than ALV at preventing HCV and HIV-1 infections in mice. (Lim et al., 2011). As with NV556, rencofilstat has been demonstrated to be preventative in mouse models of MASLD/MASH, including HCC. (Kuo et al., 2019a). Recently, rencofilstat has also been shown to be anti-tumorigenic in HCV-induced HCC models independently of its anti-viral properties (Stauffer et al., 2023), suggesting Cyp inhibition may be effective as a direct cancer treatment beyond merely addressing the causative diseases. It is currently the most clinically advanced CypI for the treatment of metabolic liver disease. It is now in Phase IIa clinical trials in patients with NASH with advanced fibrosis (F2/F3). (Hepion Pharmaceuticals, 2024). It will be interesting to determine whether rencofilstat treatment will provide similar or superior beneficial effect than resmetirom, an oral liver-targeted thyroid hormone receptor β (THR-β) selective agonist that showed statistically significant NASH resolution and improvement in liver fibrosis by at least one stage. (Harrison et al., 2024).

#### 3.1.7 Cyclophilin inhibitors (CypI) unrelated to CsA and sanglifehrins

By means of a fragment-based drug discovery approach based on X-ray crystallography and nuclear magnetic resonance, the Pawlotsky lab generated a new family of nonpeptidic small-molecule cyclophilin inhibitors (SMCypIs) unrelated to CsA or sanglifehrins, with potent inhibitory activity against CypA, CypB, and CypD. These compounds lack cellular toxicity and immunosuppressive activity and bear druggable pharmacologic properties. This new family of small molecule, SMCypIs, binds CypD and inhibits its PPIase activity and that this effect is partly responsible for their concentration-dependent inhibitory effect on Ca^2+^-induced swelling due to mPTP opening in both human and mouse hepatocytes. Among these new SMCypI, one compound exerts an additional effect on mPTP opening, independent of its inhibitory effect on CypD, making it a promising pharmacologic agent for liver damage protection in the context of diseases involving mitochondrial dysfunction related to abnormal mPTP opening. The Pawlotsky lab provides the *in vivo* proof-of-concept of mitochondrial and liver damage protection by SMCypI in an experimental model of ischemia/reperfusion injury. (Panel et al., 2019).

The Liang lab via a structure-based *in silico* virtual screening followed by surface plasmon resonance and a PPIase inhibition assay to identify cyclophilin J (CypJ) inhibitors. They optimized anti-tumor cell line proliferation activity of CypJ inhibitors using quinoxaline nucleus-associated rational design. Among them, one compound with a quinoxaline nucleus, showed potential for tumor cell line proliferation inhibition, suggesting that these small molecules may represent novel potential lead compounds for CypJ-based antitumor drug development. (Zhao et al., 2018).

The Liu lab identified new CypI from an *in vitro* selection of a DNA-templated library of 256,000 drug-like macrocycles for CypD affinity. Repeated macrocycle engineering guided by X-ray co-crystal structures yielded potent and selective inhibitors that bind the active site of CypD. The resulting macrocycles inhibit CypD activity and mPTP opening in isolated mitochondria. [ (Peterson et al., 2022)]

### 3.2 Diseases in which cyclophilin inhibitors (CypI) have been clinically investigated as therapeutics

#### 3.2.1 Chronic viral hepatitis

Hepatitis C is a common form of viral hepatitis (liver inflammation) caused by infection with HCV. HCV is from the *Flaviviridae* family of enveloped, (+) single-strand RNA viruses (Figure 2). HCV is spread by blood-to-blood contact, most commonly by contaminated needle sticks, needle sharing, and blood transfusions. It affects tens of millions of people worldwide and accounts for approximately 290,000 deaths per year from complications related to liver failure, cirrhosis, or HCC. There are currently six HCV genotypes (1–6) and no vaccine. (Lauer and Walker, 2001; Manns et al., 2017; Manns and Maasoumy, 2022). The immunity against a particular HCV genotype does not protect against subsequent infections with a different HCV genotype. The efficacy of drug treatments varies from genotype to genotype and thus it is important to know the HCV genotype of each patient before prescribing treatment. The availability of direct-acting antiviral agents (DAAs) for the treatment of hepatitis C has greatly expanded over the last decade. At the time ALV was undergoing clinical trials for HCV infection the SOC was a combination of PEG-IFNα2a, a synthetic IFN which activates IFN-stimulated genes and enhances the host immune system, and ribavirin, a nucleoside-analog prodrug which interferes with viral RNA replication, causing a high rate of mutations. (Pawlotsky, 2013). Though this drug combination continues to be commonly used, efficacy is limited and side effects can be severe. (Baugh et al., 2013). More recently drugs such as NS5B inhibitor sofosbuvir (Bhatia et al., 2014), NS5A inhibitors daclatasvir (McCormack, 2015) and ledipasvir (Scott, 2018), and NS3/4A inhibitor simeprevir (Izquierdo et al., 2014) have been FDA approved and are used frequently in combination with each other or with the earlier IFN/ribavirin SOC to treat different HCV viral genotypes. Combination therapies are often used to overcome viral mutations leading to drug resistance, though efficacy can vary depending on the HCV genotype in question and whether there is co-infection with HIV-1. (Laiwatthanapaisan and Sirinawasatien, 2021). A further issue is the cloaking of the viral genome to the host innate immune system through the formation of viral replication organelles (ROs), double-membrane vesicles (DMVs) where viral genome synthesis occurs in an optimal environment, using viral proteins and host factors. (Paul et al., 2013). Current treatments also do not treat downstream consequences of hepatitis C, such as liver fibrosis or HCC formation. CypI have been repeatedly shown to have potent anti-HCV potential through multiple mechanisms, mostly centering on the binding and inactivation of CypA. CypA is known to complex with NS5A, a necessary step for the formation of viral ROs. CypA-NS5A interactions control the formation of DMVs that protect the newly synthesized viral genome from recognition by immune host sensors. (Mamatis et al., 2022). Furthermore, CypA is a master regulator of the innate antiviral immune response through multiple avenues. It physically interacts with RNA-dependent protein kinase R (PKR), preventing its autophosphorylation and its downstream promotion of interferon stimulated genes (ISGs). (Bobardt et al., 2014; Daito et al., 2014; Colpitts et al., 2020). ISG expression can be restored via the CypI SCY635. (Hopkins et al., 2012b). Conversely, CypA promotion of RIG1 ubiquitylation by TRIM25 has been shown to be necessary for RIG1-mediated antiviral immune activation, highlighting the often contradictory roles of ubiquitous proteins such as CypA. (Liu et al., 2017). CypI have most frequently been investigated for their role in preventing HCV infection. The most notable CypI in this regard, as discussed above, are ALV, which advanced to Phase III trials in combination with IFN/ribavirin therapy (Zeuzem et al., 2015) and rencofilstat, which has been shown to prevent not only HCV infection in mice but also downstream HCC formation, independent of its antiviral properties. (Stauffer et al., 2023).

Viral hepatitis is also caused by other viruses unrelated to HCV, including hepatitis A virus (HAV), HBV, hepatitis D virus (HDV), and hepatitis E virus (HEV). Of these, HBV is the only other to frequently cause chronic illness. (Castaneda et al., 2021). HBV is an enveloped double-stranded DNA virus from the *Hepadnaviridae* family. Transmission occurs most often with blood contact, sexual contact, or mother to child. Due to similarity in routes of transmission, co-infection with other viruses such as HCV or HIV-1 is common. HBV vaccines provide lifetime protection, but do not eliminate the need for HBV treatments. Chronic HBV is still endemic in certain populations and can lead to cirrhosis and HCC. HBV infection elevates serum CypA levels and there are indications that CypA is involved in HBV integration and antigen secretion, though further research is needed. Non-immunosuppressive CypI ALV and NIM811 both suppressed HBV DNA and HBV surface antigens in cell culture (Phillips et al., 2015) and rencofilstat impaired HBV infection in humanized mice (Gallay et al., 2019) and has also been investigated in Phase I clinical trials in patients with chronic HBV.

#### 3.2.2 Acquired immunodeficiency syndrome (AIDS)

HIV, the causative agent in AIDS, is an enveloped, (+) sense single-stranded RNA virus from the Retroviridae family and the *Lentivirus* genus (Figure 2). Of two subtypes, HIV-1 and HIV-2, HIV-1 is the more virulent, transmissible, and widespread. Transmission is usually through sexual contact and the exchange of bodily fluids. HIV-1 primarily infects immune cells, such as CD4^+^ T-cells and macrophages, through the CD4 receptor. This causes progressive immune system collapse as HIV-1 infection progresses to AIDS, leading to death from opportunistic infections or cancer in the absence of treatment. HIV-1 replicates rapidly and has a high rate of error during reverse transcription, leading to a high rate of mutation and the potential for the generation of drug resistant strains. (Pantaleo and Fauci, 1996; Simon et al., 2006; Deeks et al., 2015; Bekker et al., 2023). Because of this, a vaccine has thus far been elusive (though an mRNA vaccine against HIV-1 has recently been through Phase I clinical trials) (Fortner and Bucur, 2022) and drug therapies involve the use of multiple antiviral drugs targeting different aspects of the viral lifecycle at once. Termed highly active anti-retroviral therapy (HAART), this treatment involves a combination of drugs, including: entry inhibitors targeting host cell co-receptors like CCR5 and CXCR4; nucleoside reverse transcription inhibitors which can halt the conversion of viral RNA into DNA via chain termination; non-nucleoside reverse transcription inhibitors which act on the viral reverse transcriptase by binding it directly; direct inhibitors of the viral integrase enzyme, which integrates viral DNA into the host genome; viral protease inhibitors which impair the production of mature virion particles upon budding from the host cell. (Maartens et al., 2014; Cihlar and Fordyce, 2016; Landovitz et al., 2023). Continuous treatment with HAART has been widely successful at preventing HIV-1 infection from progressing to AIDS, transforming the infection into a manageable chronic illness. Despite this, challenges remain in combatting drug resistant mutations, highlighting a need for antiviral drugs which act on multiple aspects of the viral life cycle with minimal side effects. Additionally, patients from at-risk groups, such as IV-drug users, may be co-infected with other pathogens. For example, as previously noted, coinfection with HCV and HIV-1 is a common circumstance. (Taylor et al., 2012; Chen et al., 2014). Drugs which can address both infections at once would have obvious benefits in these cases. Cyp inhibition has intriguing potential to address these concerns. When CsA was originally found to prevent HIV infection of T cells, it suggested that CypA may play a role in HIV replication. (Sokolskaja et al., 2010). CypA is now known to directly bind the HIV-1 gag protein and the capsid protein where it stabilizes the capsid.[ (Taylor et al., 2012; Kim et al., 2019)] CypA has been demonstrated to variously participate in viral uncoating (Luban, 1996), reverse transcription (De Iaco and Luban, 2014), nuclear import (Schaller et al., 2011), and may help shield the viral genome from host restriction factors (Liu et al., 2016; Selyutina et al., 2020). CypB has also been found to bind the gag protein and to influence HIV-1 infectivity. (Luban et al., 1993; Chertova et al., 2006; Ambrose and Aiken, 2014). These roles began to be elucidated relatively early in the HIV-1 epidemic when CsA was shown to prevent HIV-1 infection in certain cultured immune cells. (DeBoer et al., 2016). Similar subsequent studies in non-immunosuppressive CsA analogs have found that these also potently abolish HIV-1 infectivity showing that CypI anti-HIV-1 effects are independent of CsA’s immunosuppression. (Rosenwirth et al., 1994; Billich et al., 1995; Gallay et al., 2015). In particular, ALV has undergone Phase I clinical trials in HIV-1 and HCV co-infected patients and shown, as a monotherapy, to reduce the viral load from both viruses. This first clinical trial represented a milestone in the HCV treatment field since the original goal of the trial was to determine the inhibitory potency of ALV against HIV-1 but it showed somewhat unexpectedly that ALV had a far greater potency against HCV than HIV-1. (Wainberg et al., 1988). Rencofilstat has shown similar potential against co-infection in cell culture. (Gallay et al., 2015).

#### 3.2.3 Coronavirus disease 2019 (COVID-19)

COVID-19 illness and the associated ongoing global pandemic are caused by SARS-CoV-2, a (+) sense, single-stranded RNA virus of the Coronaviridae family. SARS-CoV-2 has many strains, many of which continue to emerge, though all are transmitted via aerosolized droplets and use their spike protein to bind with the ACE2 receptor in respiratory epithelial cells. (Flisiak et al., 2008; Lamers and Haagmans, 2022). CypA has been shown to bind the spike and nucleocapsid proteins of SARS-CoV-2 and other coronaviruses with high affinity. (Hu et al., 2021). There, evidence suggests it facilitates virus-host cell interactions and viral entry. This may also involve interactions between CypA and its well characterized binding partner CD147. (Sheng et al., 2023). As with other coronaviruses, While various CypI compounds are able to prevent infection with other coronaviruses in cell culture (Tanaka et al., 2013; Carbajo-Lozoya et al., 2014; Liu et al., 2020; Sauerhering et al., 2020), evidence of CypI prevention of SARS-CoV-2 infection is limited to one study (Softic et al., 2020). This was enough to advance ALV to Phase II clinical trials for the treatment of patients with established COVID-19 during the height of the COVID-19 pandemic but trials have not advanced since then. However, the broad-spectrum antiviral characteristics of CypI remain appealing in light of the ongoing appearance of new strains of the virus.

#### 3.2.4 MASLD/MASH

**FIGURE 3:**
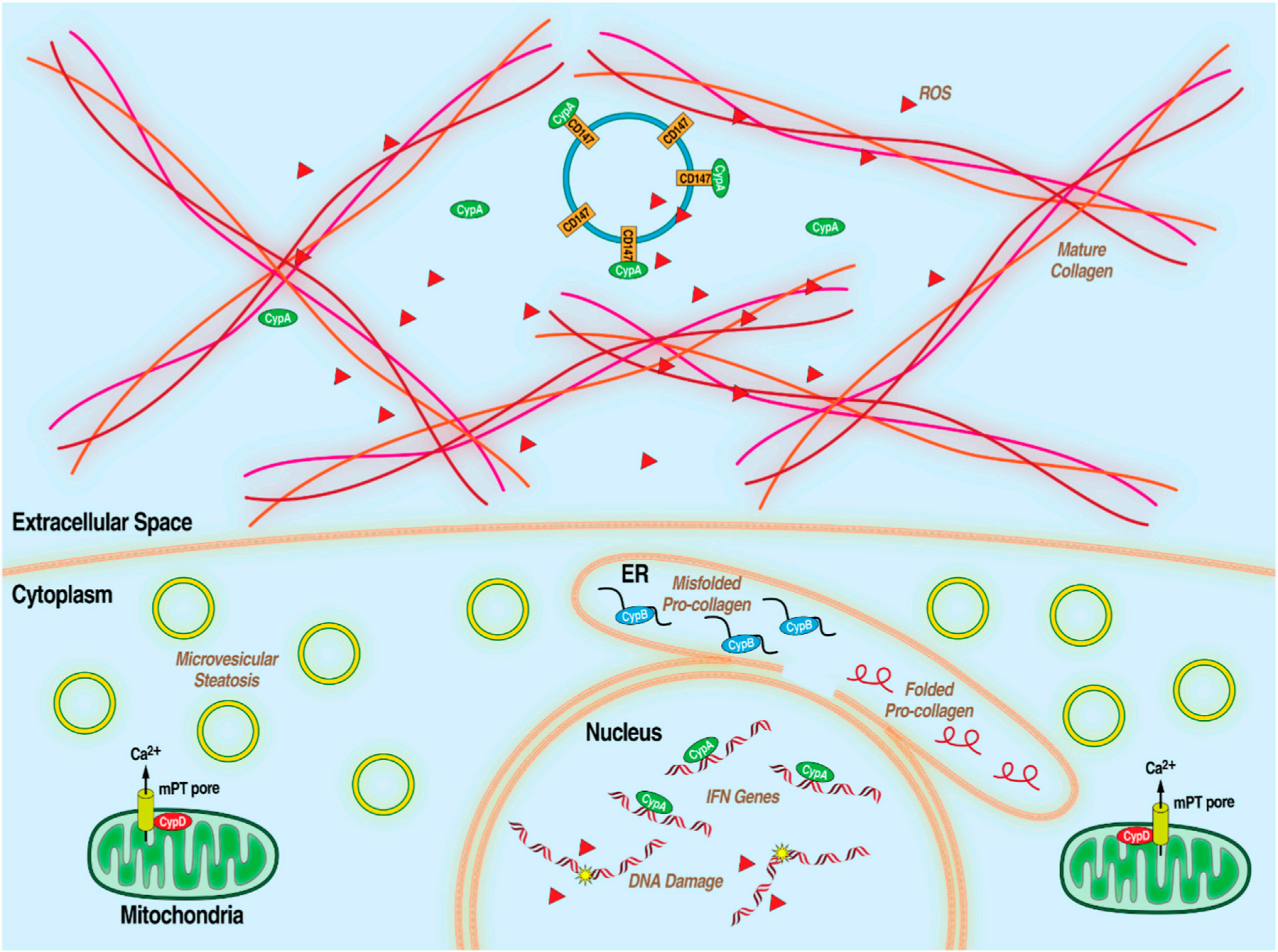
Diagram of MASLD/MASH at the hepatocellular level with the processes that Cyps have been demonstrated to be essential.

MASLD, formerly non-alcoholic fatty liver disease or NAFLD, is a condition featuring steatosis–lipid accumulation–in liver hepatocytes in the presence of metabolic risk factors but without excessive alcohol consumption or other liver toxicity. It is heavily conflated with poor diet, lack of exercise, obesity, diabetes and the wider metabolic syndrome (MetS), which is an array of factors associated with metabolic dysfunction, including dyslipidemia, hypertension, and insulin resistance. Early stages are generally symptom-free and diagnosis is only definitively made with invasive liver biopsy so MASLD often goes undetected until it progresses to its more advanced form, MASH, formerly NASH which is characterized by chronic liver inflammation, leading to hepatocyte death and, often, liver fibrosis. Though MAFLD and MASH in its earlier stages are reversible with lifestyle changes, extensive MASH-related fibrosis can progress to cirrhosis and severely impaired liver function. (Anstee et al., 2013; Bellentani, 2017; Fernando et al., 2019; Loomba et al., 2021; Younossi et al., 2023). Chronically inflamed liver tissue is also high in oncogenic reactive oxygen species leading to development of HCC. (Balogh et al., 2016; Chen et al., 2019). MASLD is extremely common, affecting 1 in 4 people. HCC is the most common primary liver cancer and MASH-related cirrhosis is the leading cause of liver transplantations after HCV infection. Despite the wide spread of these related diseases, there is currently no approved treatment for MASLD/MASH beyond lifestyle changes and weight loss. (Dufour et al., 2022; Machado, 2023). Because of the complexity of the disease, broad-acting therapeutics are needed to address its disparate aspects. Hints at the importance of Cyps in the development of multiple aspects of MASLD/MASH have been accumulating over the last 2 decades (Figure 3). For example, extracellular CypA interacts with the CD147 receptor to mediate chemotaxis of leukocytes, monocytes, and macrophages, thus contributing to inflammation. (Jin et al., 2004; Yang Y. et al., 2008; Yurchenko et al., 2010; Phillips et al., 2015). CypB, through its role as an endoplasmic reticulum chaperone for proteins exported down the classical secretory pathway, is required for collagen export to the extracellular medium where it forms one of the primary components of fibrotic scarring. (Choi et al., 2014; Khong et al., 2020). Several lines of evidence suggest that CypD plays an important role in liver damage including liver fibrosis (MASH) and HCC. As part of the mPTP, CypD is an important part of cell death pathways and its inhibition is protective in some tissues. (Elrod et al., 2010; Sui et al., 2018). CypD promotes cancer cell death by binding and inactivating the tumor suppressor p53 (Machado, 2023). Supporting the notion that CypD participates in the development of liver damage, non-immunosuppressive CypI such as the sangamide NV556 (Kuo et al., 2019b) and the CsA-analog rencofilstat (Kuo et al., 2019a) decreased liver fibrosis in diabetes-linked mouse models of MASH. Moreover, both CypI also significantly decreased the formation of HCC tumors. Since sangamide and CsA-derivatives potently bind and inactivate all tested Cyp family members, it is reasonable that Cyp isoforms (i.e., CypB and CypD) are essential for the progression of MASLD/MASH. This further highlights the potential for Cyp inhibition as a strategy to address the distinct features of complex diseases. Rencofilstat has advanced to Phase II clinical testing for MASLD/MASH (Harrison et al., 2022).

## 4 Conclusion

While no drug treatment can be considered a cure-all, there are advantages in seeking broad-spectrum compounds which can treat a variety of conditions such as CypI. Another advantage of compounds that provide beneficial effect against multiple human diseases is the significant reduction in the number of medications that patients must take. Such compounds are difficult to develop since they would need to influence the extremely varied cellular processes involved in otherwise unrelated diseases. The common denominator is therefore likely to be of such importance to cellular function that perturbing it via a drug seems likely to be toxic. The Cyp family of PPIases however seems to be the rare instance of a highly conserved, ubiquitously expressed group of proteins involved in a wide range of important functions which nevertheless can be inhibited or knocked out without severe consequences at the tissue or organismal level. KO mouse models of the three most studied Cyps - CypA, CypB and CypD - show that mice are viable and useable for research despite some reported abnormalities. (Machida et al., 2006; Choi et al., 2009; Eliseev et al., 2009; Nederlof et al., 2017). It is important to emphasize that at this stage, we cannot exclude the possibility of redundancy or compensatory mechanisms induced when a single Cyp isoform is KO in animals. A similar observation can be made with CypI treatments since they either may neutralize all Cyp isoforms or more preferentially specific Cyp isoforms. CypI in clinical trials have repeatedly been shown to be relatively well tolerated. The one Phase III trial involving a CypI, ALV, was placed on hold due to three acute cases of pancreatitis, including one death, but it was never formally shown whether these cases were due to ALV itself or to the combination of IFN and ribavirin therapy. (Zeuzem et al., 2015). To date, severe pancreatitis has never been observed with monotherapies of ALV or other CypI. Currently there are multiple active Phase 2 clinical trials investigating rencofilstat as a monotherapy for the treatment of MASH, especially in patients with advanced (F3) liver fibrosis. The ability to treat already established liver disease is key with MASH especially since diagnosis is typically not made until a more advanced disease state. Cyp inhibition has shown promise in reducing the infectivity of numerous unrelated viruses, in ameliorating fibrotic disease, and even as a tumor suppressor. A drug class with therapeutic potential for such a wide variety of conditions is deserving of further investigation.
